# An Outbreak of Human *Fascioliasis gigantica* in Southwest China

**DOI:** 10.1371/journal.pone.0071520

**Published:** 2013-08-08

**Authors:** Jia-Xu Chen, Mu-Xin Chen, Lin Ai, Xue-Nian Xu, Jian-Ming Jiao, Ting-Jun Zhu, Hui-Yong Su, Wei Zang, Jia-Jun Luo, Yun-Hai Guo, Shan Lv, Xiao-Nong Zhou

**Affiliations:** 1 The National Institute of Parasitic Diseases, Shanghai, the People’s Republic of China; 2 Key Laboratory on Biology of Parasite and Vector, Ministry of Health, People’s Republic of China, WHO Collaborating Center for Malaria, Schistosomiasis and Filariasis, Shanghai, People’s Republic of China; 3 The Affiliated Hospital of Dali University, Dali, the People’s Republic of China; 4 The People’s Hospital of Dali Prefecture, Dali, the People’s Republic of China; 5 Dali Institute of Schistosomiasis Control, Dali, the People’s Republic of China; The Australian National University, Australia

## Abstract

Fascioliasis is a common parasitic disease in livestock in China. However, human fascioliasis is rarely reported in the country. Here we describe an outbreak of human fascioliasis in Yunnan province. We reviewed the complete clinical records of 29 patients and performed an epidemiological investigation on the general human population and animals in the outbreak locality. Our findings support an outbreak due to *Fasciola gigantica* with a peak in late November, 2011. The most common symptoms were remittent fever, epigastric tenderness, and hepatalgia. Eosinophilia and tunnel-like lesions in ultrasound imaging in the liver were also commonly seen. Significant improvement of patients’ condition was achieved by administration of triclabendazole®. *Fasciola* spp. were discovered in local cattle (28.6%) and goats (26.0%). Molecular evidence showed a coexistence of *F. gigantica* and *F. hepatica*. However, all eggs seen in humans were confirmed to be *F. gigantica*. Herb (*Houttuynia cordata*) was most likely the source of infections. Our findings indicate that human fascioliasis is a neglected disease in China. The distribution of triclabendazole®, the only efficacious drug against human fascioliasis, should be promoted.

## Introduction

Fascioliasis, due to *Fasciola hepatica* and *F. gigantica,* is an important parasitic disease with a particular concern in animal health [1], [2]. More than 700 million domestic animals are at risk all over the world and economic loss exceeds US$ 2 billion per year [3]. Traditionally, human fascioliasis is rarely considered and reported; the estimated number of cases was less than 3000 before 1990. However, recent estimates indicate that approximately 2.4 million people in more than 60 countries are infected [4] and over 180 million live at risk of fascioliasis [5]. A global analysis of the geographical distribution of human cases shows that the expected correlation between animal and human fascioliasis only appears at a basic level [6]. Nowadays, world trade, climate change and population movement are important indicators challenging infectious diseases [7], [8].

Only 44 human cases were documented in China before 1990 [9]. An additional 49 sporadic human cases were reported in the following two decades. That human fascioliasis is neglected is obvious, since the total estimate of infected humans amounted to 120,000 according to the first national survey on the major parasitic diseases in 1992 [10]. As in other countries, effective diagnosis, treatment are challenging the control of fascioliasis. Stool examination and serological precipitation methods are choices for parasitological diagnosis. However, the detection of *Fasciola* eggs in stool samples is not easy in the early stage of infection or at low infection levels [11]. The only effective drug, triclabendazole®, has not yet been registered in China [12].

Although *F. hepatica* and *F. gigantica* have different geographic distributions [6], both species essentially share the same life cycle. Livestock and humans are common definitive hosts, while wild mammals may serve as reservoirs [13], [14]. Adult worms can survive up to twenty years in the definitive host. Its body length ranges from 13.4 mm to 38. 5 mm and the width from 5.6 mm to 13.4 mm [15]. However, the longevity and worm size vary [15], [16]. Adult worms reside in the biliary system and cause chronic inflammation and obstruction eventually leading to cholangitis, cholecystitis and cholelithiasis [11]. However, the most important effect has to do with the early larval migration in the liver causing hepatic lesions, which result in fever, abdominal pain, hepatomegaly. Before they mature in the biliary ducts, the larval forms can migrate for 10–12 weeks in the liver [11], [16]. Therefore, the acute syndrome due to the lesions may last for several months. The eggs are released with the bile and expelled with the feces. The hatched miracidia invade freshwater snails (Lymnaeidae) and develop into cercariae, which encyst as metacercaria after sticking to aquatic plants. Human and mammals become infected when ingesting raw, contaminated plant material. The metacercariae excyst and invade the intestine wall to enter the peritoneal cavity, where the flukes home in on and penetrate the liver parenchyma, thus closing the cycle.

Although fascioliasis caused by *F. gigantica* may share a number of similarities with that caused by *F. hepatica* clinically and epidemiologically, systematic studies of fascioliasis outbreaks due to *F. gigantica* have rarely been carried out. This report provides the clinical profile and the epidemiological features of an outbreak and is expected to fill the knowledge gaps in surveillance and response to food-borne diseases in China.

## Methods

### Ethical Statement

Ethical clearance for the collection and examination of human sera or feces samples was obtained from the Ethics Committee of the National Institute of Parasitic Diseases (NIPD), Chinese Center for Disease Control and Prevention (China CDC). The objectives, procedures and potential risks were orally explained to all participants. The written informed consent was given to, and signed by all participating in the study. Parents/guardians provided consent on behalf of all child participants. The study did not involve the specific permission for the collection of intermediate host snails, which are not an endangered species. The collection of feces samples of livestock was permitted by the owners. No livestock was sacrificed. The animals were separated individually for collection of feces overnight. All animals were handled in strict accordance with good animal practice according to the Animal Ethics Procedures and Guidelines of the People's Republic of China, and the study was approved by the Animal Welfare & Ethics Committee of NIPD, China CDC.

### Hosptalized Patients and Clinical Information

Since mid December, 2011, a series of human cases who complained of fever and hepatalgia were admitted to local hospitals in Yunnan province. The patients were suspected to be infected by helminths, but broad-spectrum antihelminthic drugs failed to control the illness. For the sake of convenient management, the local health authorities appointed three hospitals and one local centre for parasitic diseases for further diagnosis and treatment. Thus, we collected the clinical information from the hospitals. The case was defined as follows: (1) patients who were hospitalized since December, 2011; (2) patients with fever (>37.8°C); (3) patients with eosinophilia (>500 cells/mL or 5% in differential count in the peripheral blood); and (4) patients showing hepatic lesions (e.g. hepatomegaly, hepatalgia, epigastric tenderness, or lesion by ultrasongraphy). All of the patients provided written informed consent before enrollment. They were followed for one month after discharge from the hospital.

The demographic information and their treatment history of patients before being admitted to the appointed hospitals were extracted. Physical examination results and the chief complaints were documented. In the meanwhile, we reviewed the daily records and extracted any results pertaining to biochemical and cellular tests, imaging examinations (B-mode ultrasonography and computerized tomography), axillary temperature every four hours, special interventions with anthelminthic drugs and/or hormones. Other particularly important data, such as differential diagnosis, bone marrow aspiration and examination, hepatic biopsy, and the dynamics of symptoms, were also recorded.

### Additional Examinations for Hospitalized Patients

We performed serological assay and parasitological examination on the hospitalized patients. The patients’ peripheral blood and stool samples were investigated over a few consecutive days. For the serological testing, we extracted the crude antigens from adult worms of *F. hepatica* and *F. gigantica* isolated from infected cattle and then connected the antigens to panels according to Ishida et al. [17] and Chen et al. [18]. Briefly, worms washed in phosphate-buffered saline (PBS, pH7.4) were individually homogenized with a tissue homogenizer for 3 minutes. Homogenized samples were sonicated at 13 Hz for 10 seconds and 25 cycles. After being incubated at 4°C overnight, the sonicated homogenate was centrifuged at 10,000 rpm for 30 min at 4°C. The supernatant was gathered and protein concentration was measured by the Lowry method [19] and the liquid was stored at -80°C until used. Each well of polystyrene 96-well plates was sensitized using a standardized quantity (1 mg) of crude antigen, diluted in 100 ml 0.05 M bicarbonate buffer (pH 9.6) and incubated at 4°C overnight. The plates were manually washed three times with PBS containing 0.05% tween-20 (PBST), followed by blocking with 1% (v/v) bovine serum albumin (BSA) in PBST (BSA-PBST) at room temperature for 2 h. The coated panels were used in enzyme-linked immunosorbent assay (ELISA) following the protocol [20]. The positive cutoff value was calculated as the mean optical density (OD) value of the normal controls plus 3 standard deviations (SD) [21].

As for the parasitological examination, the Hoffman-Pons-Janer method was employed [22]. Briefly, a sample of 20∼30 g stool was diluted and blended with tap water. The dregs were discarded after filtering by a 0.25 mm×0.25 mm mesh. The filtrate was allowed to stand for 30 min, after which the supernatant was discarded. The sediment was washed with tap water three times and examined for *Fasciola* spp. eggs under the microscope at 40× magnification. Eggs of *Fasciola* were first identified based by morphology described by Valero et al. [23]. All *Fasciola*-like eggs found were then confirmed by molecular tools.

### Extended Investigations

In addition to hospitalized patients, we also conducted an extended epidemiological investigation, involving the general human population, livestock, and freshwater snails in the same area where the patients lived. We investigated the prevalence of fascioliasis in humans and livestock by stool examination, and in the freshwater snails by crashing between glass slides followed by microscopy at 40× magnification.

For humans, a 1∶100 matched sampling strategy was employed, i.e., one patient and one hundred residents above 6 years living in the same community were selected. If the number of residents in a community was less than 100, all the residents were encouraged to participate in the study. They were all informed about the procedure and aims of the project by the local authorities. Only volunteers were involved in the survey. Each participant was asked to donate 5 ml peripheral blood for serology with respect to *Fasciola* spp. The participants whose blood samples proved positive were requested to present stool samples for parasitological examination.

Thirteen out of the 20 communities covered by the region where the patients were distributed were selected at random to investigate the prevalence of fascioliasis in cattle and goat populations. All cattle and goats from the 13 communities were included in the study. Stool samples from each animal were collected and encoded. The same method as mentioned for stool examination of humans was applied.

Snails were collected from the major rivers traversing the study area, including from ditches and streams within the communities and neighborhoods where patients lived, and the ditches in the waste fields where farmers grazed their cattle and goats. Due to strong drought during the study period, many rivers and tributaries dried out. In those cases, each pond in the dried river bed was checked for snails. At least 100 aquatic snails were collected from each collecting site. The coordinates of sites were recorded by a portable GPS receiver (GPSMAP76, Garmin International Inc.). Snails were firstly identified by morphology and then were crashed individually between glass slides and checked for cercariae under a dissecting microscope. The *Fasciola* cercariae were identified by DNA sequence.

### Parasite Identification

The parasites from patients, livestock and snails were identified by a molecular approach. Around 10 *Fasciola* eggs were isolated from each stool sample and transferred to a clean tube with 20 µl water to be broken up with by a vortex procedure using glass beads (0.5 mm in diameter) for 10 min [24]. The total DNA was released from the eggs by SDS/proteinase K treatment and extracted by purifying-columns (Promega, Madison, WI, USA). The same method was applied to total DNA extraction of cercariae isolated from infected snails.

Primers targeting a nuclear DNA region comprising ITS-1, 5.8S rDNA, and ITS-2 (ITS+) [25] and a mitochondrial DNA region covering *cox*1 gene [26] were used. Polymerase chain reaction (PCR) were performed in 25 µl with 2 mM of MgCl_2_, 2.5 µM of each primer, 2.5 µl 10×r*Taq* buffer, 0.2 mM of each dNTPs, 1.25 U of r*Taq* DNA polymerase (TAKARA) and 1 µl of DNA sample as follows: an initial denaturation at 94°C for 5 min, then 94°C for 30 s; 55°C for 60 s; 72°C for 30 s for 35 cycles, followed by a final extension at 72°C for 5 min [27].

Positive amplicons were purified and presented for sequencing by the ABI 377 sequencer platform with the same primers used when performing the PCR. The obtained sequences were first aligned and any ambiguous base-pair in the sequences was subjected to repeated sequencing. The validated sequences were submitted to phylogenetic analysis. We used *Clonorchis sinensis* (FJ965385) and *Opisthorchis felineus* (JN646643) as outgroup. The typical sequences of *F. hepatica* (GU112485), *F. gigantica* (GU112459) as well as intermediate forms (GU112490) were employed in the phylogenetic analysis. Neighbor-joining (NJ) was used for phylogeny construction and performed in the PAUP 4.0 Beta 10 programme. The consensus tree was obtained after bootstrap analysis using 1000 replications with values above 50%. The comparison of the ITS–1 and ITS–2 sequences of *Fasciola* spp. from patients at variable sequence positions are shown in Table S1.

### Case-control Study

A 1∶1 matched case-control study was carried out to explore the risk factors. The controls were selected from the participants whose blood samples proved negative against antigens of *Fasciola* spp. and did not experienced corresponding symptoms within half year. Sex, age and community were matched with the patient. The difference in age of a pair was less than 2 years. A questionnaire covering the feeding activity of cattle and goats and the eating habits in the late half year 2011 was designed and administrated to both controls and patients. The major dishes with raw vegetables involved in this study included wildrice stem (*Zizania* spp.), watercress (*Nasturtium* spp.), herb (*Houttuynia cordata*), and scallion (*Alliums* pp.). Additionally, the exact coordinates of the household of patients were recorded by a hand-held global positioning instrument (GPSMAP76, Garmin International Inc.).

### Statistical Analysis and Mapping

A series of databases were constructed in Access 2003. The first database pertained to basic information of patients, including age, sex, professional, address, nationality as well as the potential relationship between patients. The second regarded biochemical indicators for liver and kidney functions and electrolytes, while the third included the peripheral blood indicators. The fourth contained the records of the results of important examinations (such as CT, B-ultrasound, bone marrow aspiration) and follow-up of symptoms and signs from daily records. The fifth database recorded the chemotherapy history with respect to anti-parasitic drugs and hormones.

In addition to the database regarding basic information and clinical data, the data produced from epidemiological investigations were entered into separate constructed databases. They included participant and community code, participant name, age, and the results of serological testing and stool sample examination. The data pertaining to the stool examination of cattle and goats and the crash examination of snails were entered into two separate databases. For the case-control study, we constructed a database using Epi Info 7 (CDC, Atlanta, GA, USA). The data were double-entered.

The statistical analysis was performed in SAS 9.1 (SAS institute Cary, NC, USA). Ten variables were considered in the risk analysis in the case-control study (Table S2). We noted that two different types of *H. cordata* were consumed locally, i.e. shoot with leaves and bare roots. We hence separated the two types as independent variables. We did three analyses, i.e. univariable analysis (chi-square test), stratified analysis, and conditional logistic regression. Since the theoretical number is less than 5 in some cells, Fisher’s exact test was employed in the univariable analysis. We carried out stratified analysis for four variables, i.e. Q1, Q2, Q3 and Q9, to explore the potential confounding. Conditional logistic regression was done among the potential risk factors. All the ten variables were included and a stepwise method was employed to select the varibales. The Chi-square test was used in sero-prevalence between the patients’ family members and other potentially implicated persons. The ELISA OD values for *F. gigantica* and *F. hepatica* in patients were compared by a matched t-test. The statistical significance level in each analysis was 0.05.

The coordinates of patients, examined domestic animals, and snail-collecting sites were mapped by ArcGIS 9.1 (ESRI Inc., Redlands, CA, USA). As for the location of cattle and goats, we integrated the results from the same community and mapped the result at the centre of community.

## Results

### Outbreak Profile

**Figure 1:**
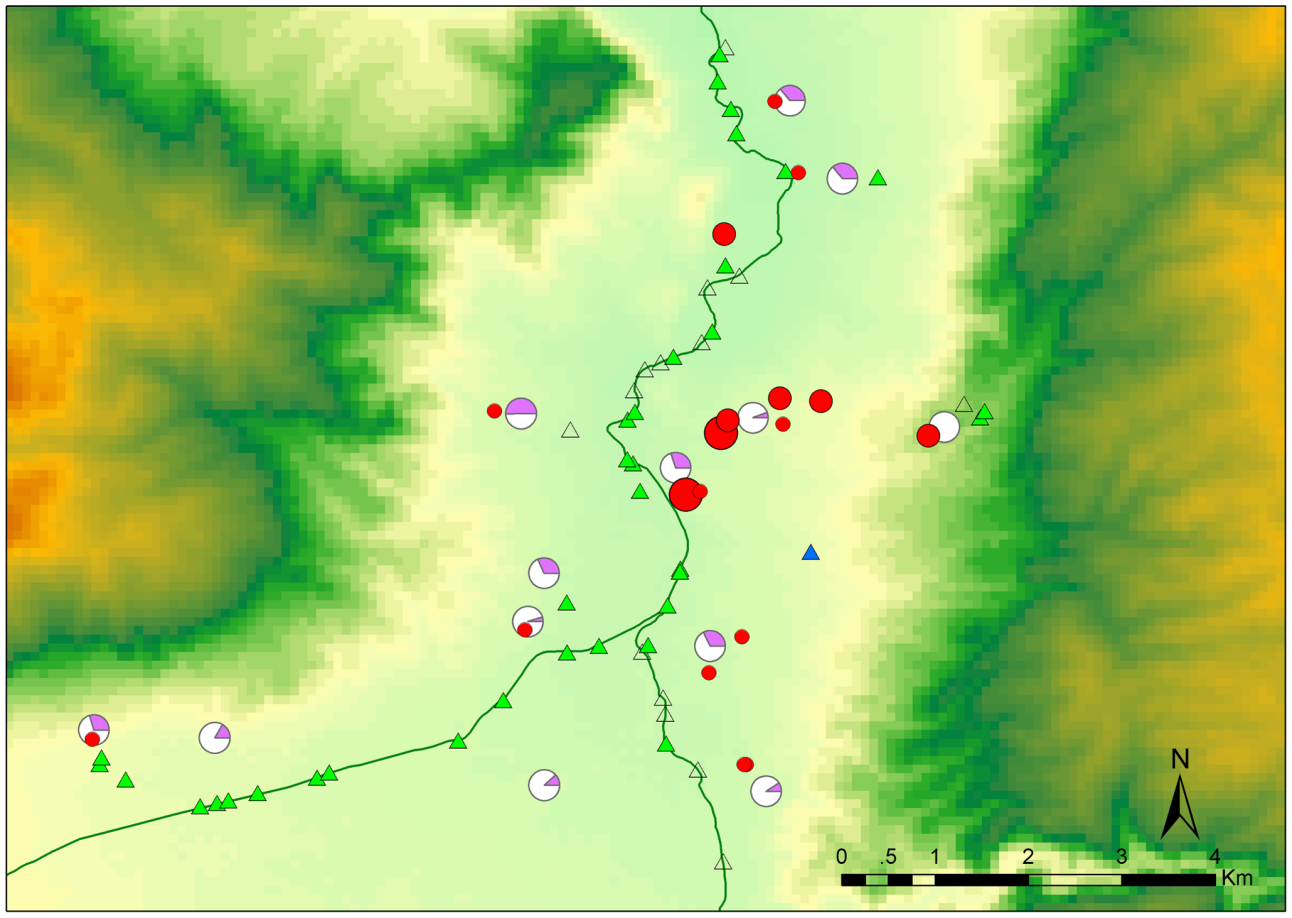
Geographical distribution of patients, cattle, goats and snails. The patients are indicated by red filled circles and the number of cases in a household is denoted by circle size. The infected cattle and goats are showed in purple share of pie chart. Triangles denote the snail-collecting sites. Open triangles indicate that no snails were observed in the water ponds, blue ones indicate that infected snails were discovered.

A total of 29 hospitalized patients from 18 families were discovered according to the definition for fascioliasis used. All the patients distributed in four adjacent townships in south part of Binchuan county, Yunnan province. Twenty eight cases were clustered within the town of Zhoucheng and neighboring communities, which are all located in a small valley (Figure 1). Two families reported four cases and five families reported two cases indicating family clustering. Only six patients from three families complained that they had dinner together in late September 2011. The other families did not report sharing dishes in the second half of 2011.

Due to insidious onset and long course of the disease, the exact time of infection was undetermined. The potential date was inferred from the clinical records and the findings from the 1∶1 matched case-control study. Figure S1 depicts the profile of the present outbreak by the two most common symptoms, i.e. fever and hepatalgia. The peak occurred during late November 2011. We also noted a second peak of hepatalgia in late January 2012. It is notable that the pain from the liver affection was generally dull and many patients were not aware of it until the condition deteriorated. Furthermore, one adult male did not report hepatalgia during the whole course of his illness.

Striking demographic characteristics were observed in these patients. Female patients accounted for 65.5% of all cases. Moreover, only two patients were less than 20 years old and the median age was 35 years. More than three quarters of all (22 cases) were farmers.

### Clinical Features

The most common signs of the infection were fever, epigastric tenderness, hepatalgia and percussion pain in the liver (Table 1). Twenty seven patients sought medical care due to intermittent fever for a few months and the remaining two became febrile after admission. Fever was moderate but might exceed 41°C in patients with fasciolosis. Hepatalgia was diagnosed in all the patients with the exception of one male adult. The pain from the liver was often dull; most patients described hepatalgia as flatulence or discomfort in the upper abdomen. Ninety percent of the patients suffered from epigastric tenderness and 62% experienced liver percussion pain. Only 10 patients showed hepatomegaly at admission and three more were found during hospitalization. Weight loss was not rare in our patients. Other less common symptoms might be directly related to fever and hepatic lesions.

**Table 1:** Manifestations of fascioliasis in 29 patients involved in an outbreak.

Symptom and sign	Admission	Whole course
Fever	27	29
Epigastric tenderness	25	26
Hepatalgia	21	28
Percussion pain in liver	18	18
Weight loss	15	15
Hepatomegaly	10	13
Feebleness	9	14
Anorexia	8	14
Cough/Expectoration	8	9
Chill	2	4
Abdominal distension	2	3
Abdominal pain	2	3
Generalized rash	1	1
Oedema	1	2
Melena	1	1
Headache/Dizziness	1	8
Muscle pain	1	4
Anhelation	1	4
Chest distress	0	4
Nausea	0	3
Vomit	0	1
Diarrhea	0	1

The most striking increase was observed in C-reactive protein. In contrast, the ratio of albumin (ALB) and globin (GLO) significantly declined. The level of alkaline phosphatase (ALP) and γ-glutamyl transpeptidase (GGT) were strongly elevated (Table S3). These findings normally imply obstructive biliary problems. However, another two indicators, i.e. bilirubin (BIL) and total bile acid (TBA), did not support this diagnosis as almost all patients showed normal total BIL and indirect BIL, while a slight increase of direct BIL was observed in 45% of the patients. Elevated TBA was found in 69% patients. Therefore, rather than an lower-level obstruction in the bilary duct, the hepatic lesions might be due to obstruction in the bile canaliculi, which would induce high ALP and GGT but not high BIL and TBA.

Alanine aminotransferase (ALT) and aspartate aminotransferase (AST) are considered as the most significant indicators of damage of the hepatic cells. Although abnormal levels were often observed, only five patients showed a constant increase in these measurements. Moreover, only two of them showed synchronous increase in ALT and AST, which implied that there were no match in the change of these two indicators. We also noted that five patients had normal ALT levels and seven normal AST levels. Therefore, the hepatic cellular damage was not as severe as implied by the manifestations observed.

The white blood cell (WBC) count generally increased in this group of patients (Table S4). With regard to the differential count of WBCs, the eosinophil proportion was almost always high. Around 48.3% of the patients showed a reduced level of red blood cells (RBCs), while 37.9% had fluctuating values. The remaining 13.8% showed normal RBC counts. Decline in haemoglobin (HGB) and haematocrit (HCT) were commonly observed in the patients. Heterogeneous shapes of red blood cells were noted in many patients (Table S4). An elevated level of platelets (PLTs) was observed, but the most of patients showed a fluctuation during the course their illness.

Bone marrow aspiration showed active proliferation in the granulocyte and megakaryocyte series. Eosinophils accounted for 20%–40% in the aspirate granulocyte series. Eosinophilic myelocyte and precursors were the predominant type. Although proliferation was also found in erythrocyte series, their proportion was slightly reduced. Orthochromatic and polychromatic normoblast were dominant. No abnormality was observed in lymphocyte or thrombocyte series.

**Figure 2:**
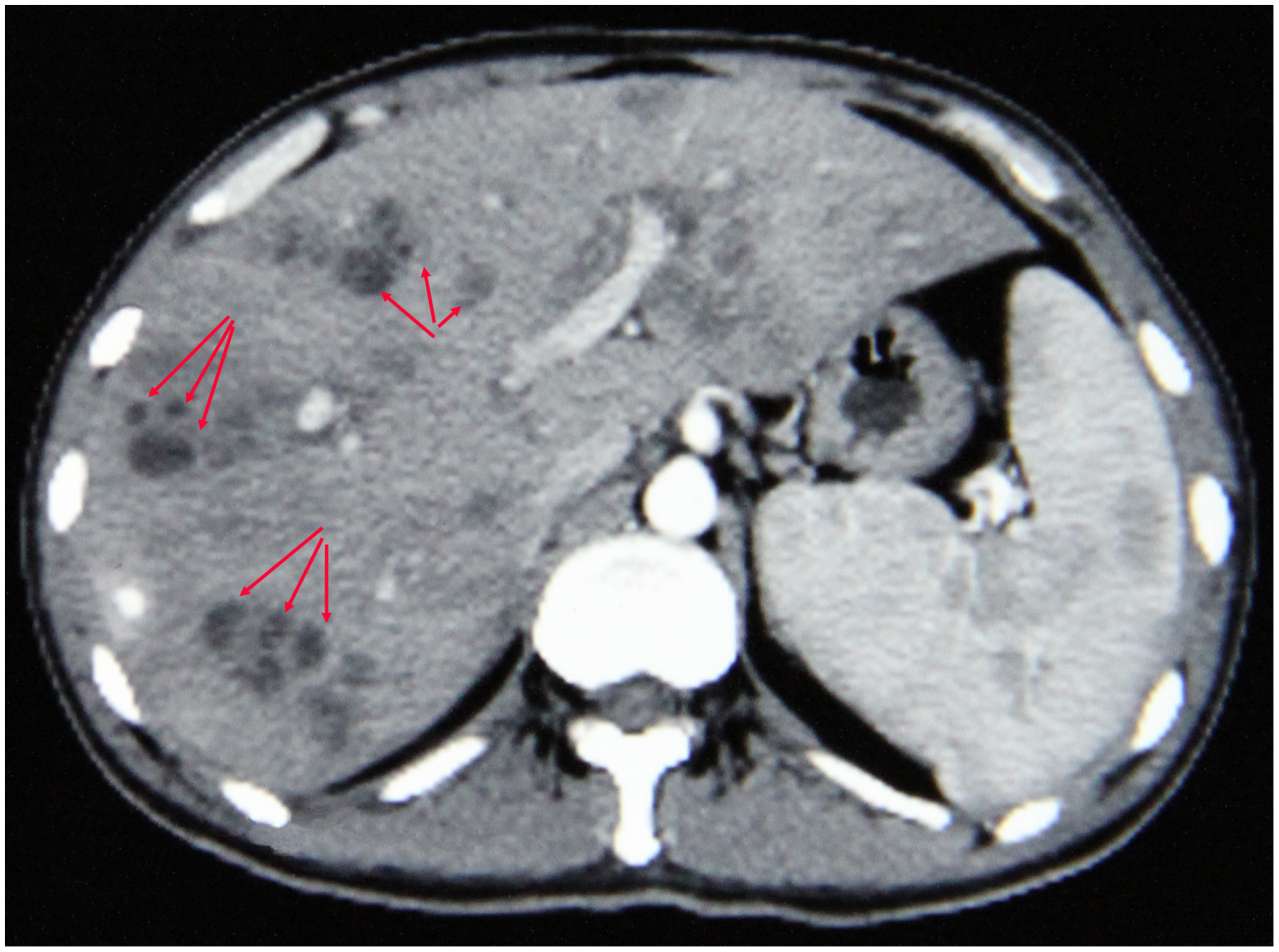
Typical image of involved liver in contrast-enhanced computerized tomography. The section shows the enlarged liver and spleen. The clusters of low density masses (arrows) in livers indicate the winding migration route of parasite.

The typical imaging of the hepatic lesions by ultrasound showed many low-density masses of irregular shapes (Figure 2). Clusters of such masses with obscure boundaries were observed in cross-sections indicating the winding migration route of parasites in the liver and the inflammatory reactions caused. These masses could occur in any lobe of liver. Splenomegaly was another common ultrasound finding: 23 out of 29 patients showed enlarged spleens. Additionally, 16 and 17 patients were found to have ascites and hydrothorax, respectively. The effusion was often small and probably due to decreased albumin levels.

In order to acquire the parasitological evidence, hepatic biopsies were performed on two severe cases during laparoscopy. Several gray convex nodes of 0.5–1.5 cm in diameter on the liver surface were observed in one patient. The microscopic sections tissue showed a mixed necrotic structure with tunnel-like lesions and small colliquative lacunae. Eosinophils, lymphocytes and plasma cells were seen around the lesions. New lesions could emerge among old ones. Proliferation of hepatic cells in areas with pathology was not active. Charcot-Leyden crystals could be observed by staining. No definite worm structures were found.

### Diagnosis and Treatment

Patients coming from the same place showed the same clinical manifestations, which inferred that they were part of an outbreak. We confirmed *F. gigantica* infection by the following evidence. First, symptoms and findings from supplementary examinations were consistent with fascioliasis. The most significant indicators included elevated eosinophil counts, clinical manifestations and typical ultrasound images plus intermittent fever. Second, a series of differential diagnosis were made to exclude bacterial and viral infections as well as other potential helminth infections (e.g. schistosomiasis, cysticercosis, angiostrongyliasis). Third, broad-spectrum anti-parasitic drugs, e.g. abendazole, mebendazole, levamisole, praziquantel and artemether, had failed to cure the illness. However, trial treatment using triclabendazole was successful in five patients. Furthermore, the eggs were confirmed as *F. gigantica* by nuclear and mitochondrial gene sequences.

**Figure 3:**
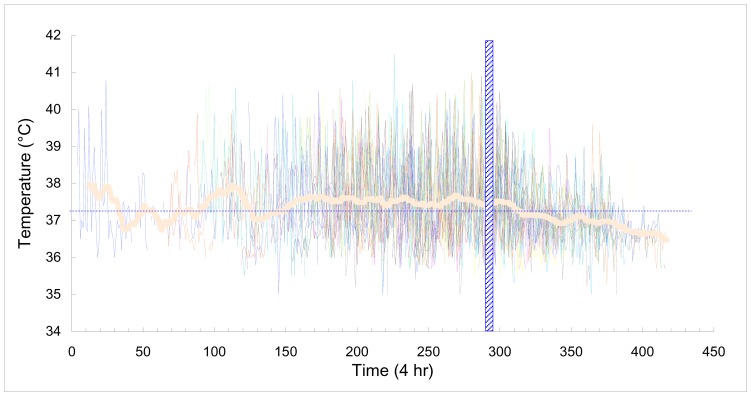
Axillary temperature profile of patients during the illness course. The vertical hatched bar indicates the treatment date using triclabendazole. The fever is defined as over 37.2°C (dash line). The thick curve denotes the average temperature, which was produced by moving average with a period of 12 (equal to 3 days).

After that the diagnosis had been confirmed, the rest of the patients were given triclabendazole using an oral dosage of 10 mg/kg/day for two successive days. Although the biochemical and cellular indicators did not become normal immediately, the patients were much improved 4–5 days post-treatment. The the elevated temperature disappeared soon and the infection was satisfactorily controlled (Figure 3). No notable side effects due to the triclabendazole medication were reported by the patients. A few patients reported a transient deterioration after chemotherapy.

We noted that a few patients complained of lipsotrichia and numbness in the lower extremities at medical follow-up one month after discharge from the hospital. However, use of other anthelminthic drugs and/or hormone treatment might might have caused the complaints. The majority of biochemical indicators reverted to normal a few weeks post-chemotherapy (Table 2). Of note, GLO, eosinophil count and the shape index of the RBCs had not recovered at this point. Although general improvement was achieved after chemotherapy, one of four parasitological-diagnosed patients continued to excrete *Fasciola* eggs 50 days post-treatment with triclabendazole, which calls for further evaluation of effectiveness.

**Table 2:** The change of major indicators after treatment with triclabendazole.

Item	SN			Indicator	Proportion of abnormal measurement		P-value
					Before	After	
*Biochemical indicators*							
	1		A/G		0.98	0.70	0.00
	2		CRP		0.97	0.04	0.00
	3		GGT		0.95	0.52	0.00
	4		ALP		0.83	0.22	0.00
	5		GLO		0.80	0.87	0.63
	6		ALB		0.75	0.00	0.00
	7		CK		0.75	0.00	0.00
	8		CHE		0.67	0.00	0.00
	9		LDH		0.62	0.39	0.04
	10		HBDH		0.56	0.39	0.16
	11		ALT		0.50	0.13	0.00
	12		AST		0.37	0.04	0.00
	13		TBA		0.28	0.09	0.05
	14		D-BIL		0.17	0.00	0.06
*Cellular indicators*							
	1	EO%			0.96	0.96	0.69
	2	EO#			0.95	0.87	0.34
	3	HGB			0.89	0.09	0.00
	4	HCT			0.89	0.13	0.00
	5	R-SD			0.76	0.87	0.26
	6	RBC			0.75	0.13	0.00
	7	R-CV			0.69	0.61	0.41
	8	WBC			0.65	0.09	0.00
	9	PLT			0.55	0.00	0.00

% and # denote the proportion and absolute count, respectively.

### Local Epidemiology

**Figure 4:**
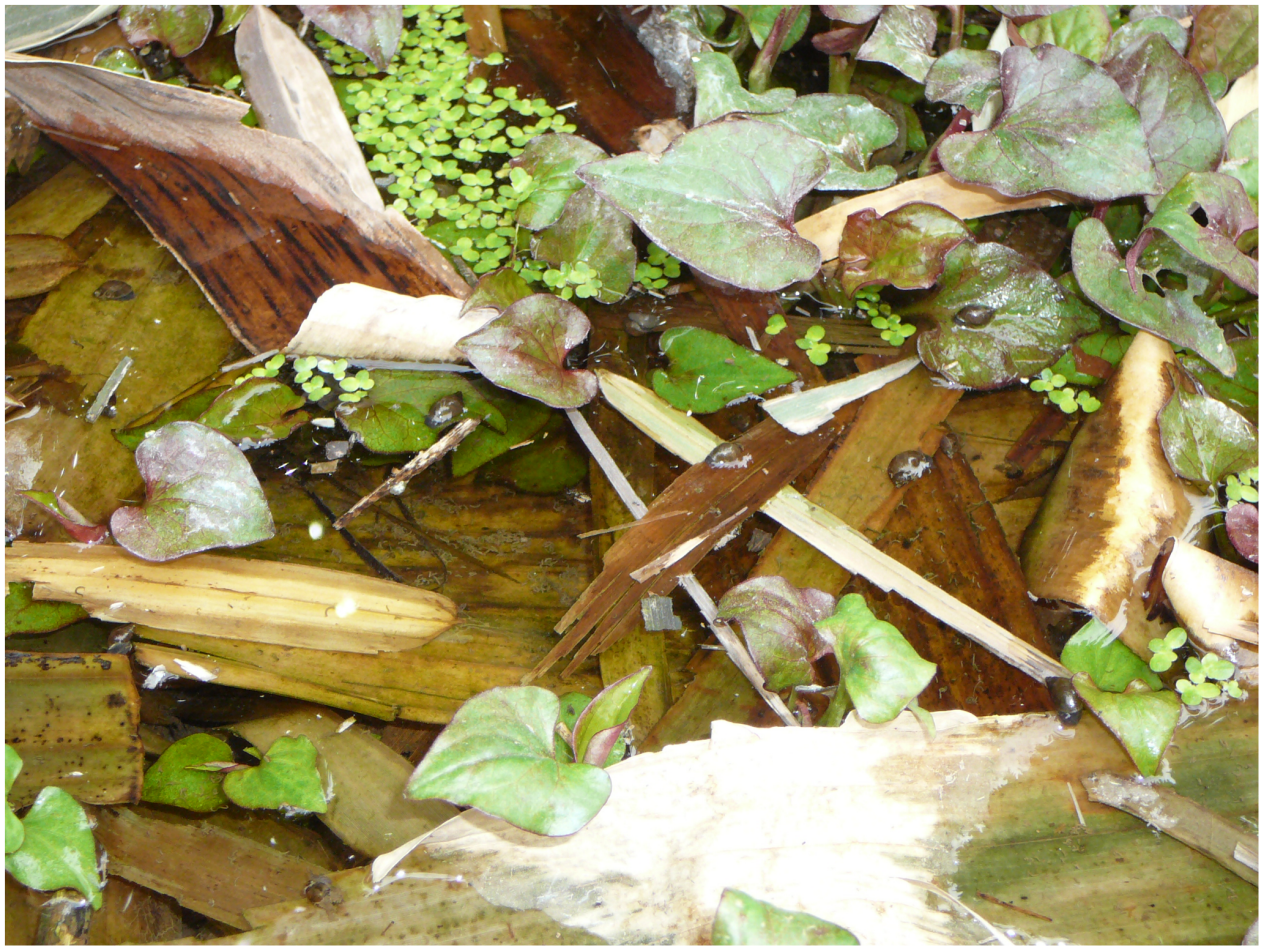
*Houttuynia cordata* growing in water. The herbs are fertilized by domestic animal faeces and *Galba* snails are stick to plants.

Water-cress, wild rice stem, a herb (*H. cordata*) and scallion were commonly consumed. Consumption of several wild vegetables was also observed. Univariable analysis showed that consumption of water-cress, wild-rice stem, *H. cordata*, and wild vegetables differed between cases and controls. Since traveling history (Q1), raising cattle (Q2), goats (Q3), and drinking (Q9) might be confounding factors, particularly with regard to the consumption of *H. cordata* shoots with leaves, these factors were considered in the multivariable analysis. Conditional logistic regression showed that only consumption of *H. cordata* shoots with leaves was significant. The Odds ratio was 2.134 and the 95% confidence interval was 1.142–3.987. We noted that *H. cordata* shoots with leaves was produced locally and normally served as a raw dish. The bare roots, in contrast, were generally imported from other places and also consumed raw but occasionally fried. Although we failed to detect *Fasciola* metacercariae from *H. cordata*, the planting pattern was seen as potentially dangerous (Figure 4). Farmers generally grow *H. cordata* in under-water fields and fertilized with faeces of domestic animals. Meanwhile, many freshwater snails (*Galba* spp.) were observed in these fields.

We obtained serum samples from 27 patients. All the samples were tested intensively against the crude antigen of *F. gigantica*. We also performed an assay for 57 family members of these patients. As a result, 26.3% participants were proven positive by ELISA. In contrast, only 8.8% (279/3177) participants beyond the patients’ family were serologically positive. The former was significantly higher than the latter (*P*<0.05), which supports a family clustering of fascioliasis. Interestingly, among the 279 serological-positive participants from the general population, only three showed *Fasciola* eggs at stool examination.

A total of 468 cattle stool samples and 104 goat stool samples from 13 communities were collected (Table S5). The overall prevalence was 28.1%. No significant difference of prevalence between cattle (28.6%) and goats (26.0%) was observed. However, the prevalence of fascioliasis in domestic animals was significantly different among these communities (*P*<0.05) and ranged from 0 to 50.7%. Of note, the prevalence in the town of Zhoucheng where 37.9% of the patients live was only 5.7%. It was obvious that less cattle and goats were bred in the town and that these animals mainly were fed in the neighboring region.

A total of 2,437 aquatic snails, belonging to three genera, i.e. *Radix*, *Physa* and *Galba*, were examined for *Fasciola* spp. Only one collecting site was inhabited by infected snails. None of 2,412 snails from the other 35 sites was found to be infected. Since heavy draught occurred in the study area during the latest three years, we failed to collect snail specimens from the communities wherefrom the patients emanated. Instead, the majority of the snails were collected from ponds in the dried river bed.

**Figure 5:**
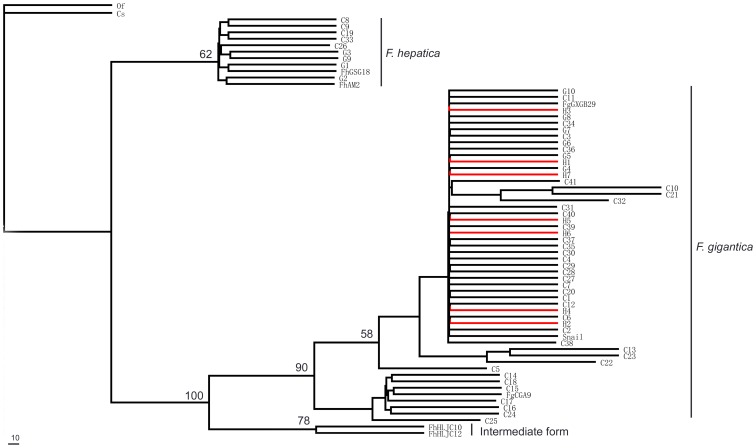
Phylogeny of *Fasciola* spp. from the present study and other sources. Red branches indicate the sequences from human stool sample. **C** represented as egg samples from cattle; **G** represented as egg samples from goat; **H** represented as eggs samples from human.

ITS+ and *cox*1 sequences of *Fasciola* spp. were obtained from 7 patients (including three cases identified in extensive epidemiological investigation), 40 cattle, 10 goats, and one snail. The sequences were aligned and truncated (399 bp for *cox*1 and 940–960 bp for ITS+). Thee phylogenetic relationship was inferred by the *cox*1 sequences. The examined specimens were clustered in two major clades (Figure 5). The big one fell into *F. gigantica* and included all the patient samples. The second one was identified as *F. hepatica* and included five samples from cattle and four from goats.

## Discussion

Fascioliasis results in significant losses, both in human health and economically. The recovery of our patients thanks to treatment with triclabendazole as a severe *Fasciola* infection is potential fatal [9], [28]. However, two key issues challenge patient management, the first of which is correct diagnosis. Presence of *Fasciola* eggs in stool is most commonly used. However, the eggs are often not discovered until 3–4 months post-infection, which is complicated by intermittent egg output dynamics [29]. The group of patients in our study was misdiagnosed at the early stage of infection, which compromised progress and resulted in severe outcomes in the majority of the patients. The second challenge is access to safe and effective drugs. All of our patients had received broad-spectrum anthelminthics without achieving any improvement. To date, triclabendazole is proven the most efficacious and best tolerated drug for the treatment of fascioliasis, which was confirmed by our findings. However, the drug is only registered in four countries, i.e. Egypt, Ecuador, Venezuela and France [12] In summary, our results highlight the urgent need to develop better diagnostic techniques and facilitate the distribution of the effective drug triclabendazole against *Fasciola* spp.

To our knowledge, this is the first outbreak in humans due to *F. gigantica* in China, although *F. hepatica* was confirmed co-existing in the same area. First, *F. gigantica* was the dominant species according to our findings in epidemiological investigation of domestic animals. Second, the higher sero-prevalence against *F. gigantica* antigens was observed in hospitalized patients. In addition, the serological reaction was more intensive to *F. gigantica* than *F. hepatica*. Third, three more human cases detected by the exploratory survey also showed *F. gigantica* infection. Finally, the eggs collected from the patients’ feaces were confirmed as *F. gigantica* eggs by nuclear and mitochondrial gene sequencing. Therefore, our clinical analysis and extended epidemiological investigations point to the outbreak of human *F. gigantica* infection.

*F. hepatica* is considered the major cause of fascioliasis in animals and humans in China; a total of 204 human cases were documented before 1999 and 95.1% of them were attributed to *F. hepatica* [10]. Notably, none was reported from Yunnan province where the present outbreak occurred but several human cases had been reported in the last decade [30]–[32]. Recent phylogenetic analysis of isolates from Yunnan province showed coexistence of *F. hepatica* and *F. gigantica* [33]. Indeed, our study also demonstrates that both species are endemic in the study area. The host specificity of these species might have given rise to different geographical distributions [6]. *F. hepatica* prefers goats as their definitive host with *Galba* snails as the intermediate hosts. In contrast, *F. gigantica* is best adapted to cattle and *Radix* snails. The different biological and ecological characteristics of host animals imply that *F. hepatica* is endemic in the highlands and *F. gigantica* in the lowlands, especially in tropical areas. However, areas where both species co-exist are observed. Altitude and seasonality might be the explanations [6]. Our epidemiological investigation found that only a few *Galba* snails were discovered in the basin (1450–1530 m) of the study area and instead the catch of *Radix* and *Physa* snails was rich. In contrast, only abundant *Galba* snails were found at a site situated above 2,000 m. There 82% of the cattle and goats were infected with *F. gigantica*. Therefore, *F. gigantica* is likely the dominant species in this area.

*Fasciola* spp. was strongly prevalent among cattle and goats in the study area and beyond in 1980s. However, thus far no human case of fascioliasis has been reported from this area. Climate and environmental change probably contributed to the emergence of fascioliasis in the study area, which is located in a small basin in a mountainous region. High temperature and dry climate have compelled the farmers to transfer from traditional agriculture to fruit production. Significantly lower precipitation in the last successive years interrupted the flow of the major rivers and dried the ponds in this area. Although this situation would decrease the risk for fascioliasis, the local eating habits could have increased the risk in the face of the draught. In contrast to the popular dish in the southwest China consisting of bare *H. cordata* roots, shoots with leaves of *H. cordata*, which are planted in under-water fields, is the most common raw dish in this area. Due to the reduced precipitation many farmers give up growing *H. cordata* and instead buy the vegetable from a few farmers. During our investigation we noted that faeces of domestic animals was used as fertilizer for the *H. cordata* fields and that there were numerous aquatic snails in the water surrounding the plants, thus facilitating the transmission of *Fasciola*. Although we could not detect larvae of *F. gigantica* from these snails, the raw dish with shoot and leaves of *H. cordata* is the most likely source of infection in this outbreak.

Our multivariate analysis showed that consumption of *H. cordata* shoot with leaves is the only significant risk factor. Indeed, the dish is frequently consumed by local people, particularly in the summer (June - September). It is well known that fascioliasis has a long acute stage or prepatent period (normally 3–4 months). According to our results the peak of this outbreak occurred in late November 2011. Hence, the time of exposure was probably during August and September. Indeed, we noted six patients from three families who complained that they had dined on raw *H. cordata* shoot and leaves during late September 2011. *H. cordata* are commonly planted in dry soil in China. Our study indicated that the raw dish with shoot and leaves of *H. cordata* growing in water is a new way of for *Fasciola* infection in our country.

*Fasciola* spp, i.e. *F. hepatica* and *F. gigantica*, are the most common parasites in ruminant animals overall the world. Death due to *Fasciola* infections in cattle and goats is common [34], [35]. Nevertheless, human fascioliasis is neglected. Our present study provides detailed information and is expected to yield new approaches for mitigating parasite transmission and virulence both in human and animal by the One Health approach, and eventually for improving the control and elimination of fascioliasis [36], [37]. Important control approaches would be health education and ending the habit of fertilizing the fields with animal feces.

## Supporting Information

Figure S1 **The profile of fascioliasis outbreak by fever and hepatalgia.**
(TIF)

Table S1 Comparison of the ITS–1 and ITS–2 sequences of *Fasciola* spp. from patients at variable sequence positions.
(DOC)

Table S2 Variables included in statistical analysis.
(DOC)

Table S3 The profile of biochemical indicators in patients.
(DOC)

Table S4 The profile of blood cellular indicators in patients.
(DOC)

Table S5 The prevalence of fascioliasis in domestic animals.
(DOC)
