# Supplementary figures and images for: An Outbreak of Human Fascioliasis gigantica in Southwest China
Source: PLoS One. 2013 Aug 8;8(8):e71520. doi: 10.1371/journal.pone.0071520 (PMC3738520; doi:10.1371/journal.pone.0071520)

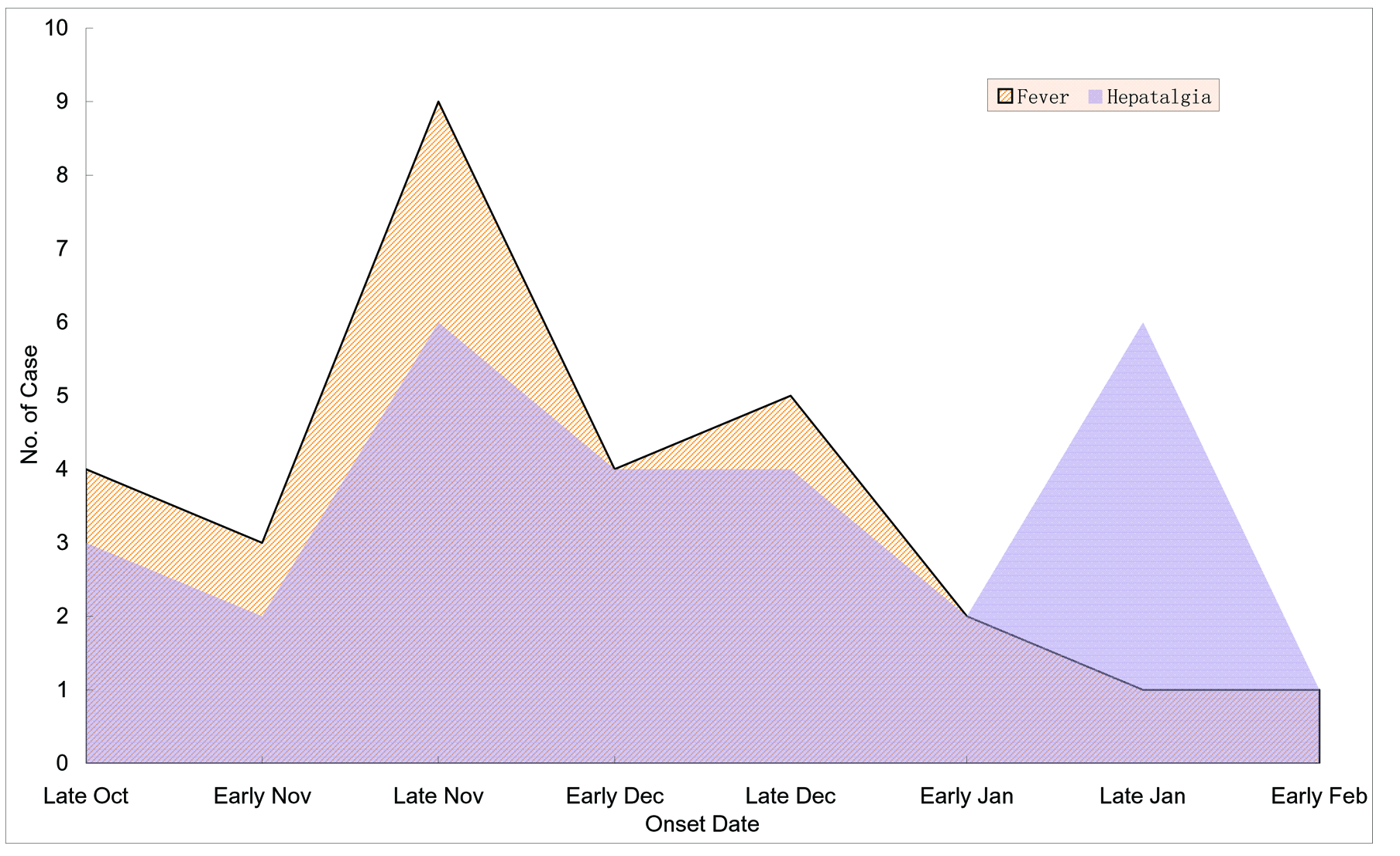

Supplement: Figure S1 — The profile of fascioliasis outbreak by fever and hepatalgia. (TIF) [file pone.0071520.s001.tif]
