# Supplementary material for: An Outbreak of Human Fascioliasis gigantica in Southwest China
Source: PLoS One. 2013 Aug 8;8(8):e71520. doi: 10.1371/journal.pone.0071520 (PMC3738520; doi:10.1371/journal.pone.0071520)
Supplement: Table S1 — Comparison of the ITS–1 and ITS–2 sequences of Fasciola spp. from patients at variable sequence positions. (DOC) [file pone.0071520.s002.doc]

Table S1 Comparison of the ITS-1 and ITS-2 sequences of *Fasciola* spp. from patients at variable sequence positions

| **Sample code** | **Variable positions in the ITS–1 and ITS–2 sequence** | | | | | | | | | | | | |
| --- | --- | --- | --- | --- | --- | --- | --- | --- | --- | --- | --- | --- | --- |
|  | **18** | **108** | **202** | **280** | **300** | **791** | **815** | **854** | **860** | **911** | **918** | **925** | **926** |
| *Fasciola gigantica* ^a^ | T | T | T | A | T | C | C | T | T | – | A | A | T |
| *Fasciola* spp. ^b^ | C/T | A/T | C/T | T/A | C/T | T | T/C | C/T | C/T | T/– | G/A | A | T |
| *F. hepatica* ^c^ | C | A | C | T | C | T | T | C | C | T | G | A | T |
| *Fasciola hepatica* ^d^ | C | A | C | T | C | T | T | C | C | T | G | T | A |
| H1 | T | T | T | A | T | C | C | T | T | – | A | T | A |
| H2 | T | T | T | A | T | C | C | T | T | – | A | T | A |
| H3 | T | T | T | A | T | C | C | T | T | – | A | T | A |
| H4 | T | T | T | A | T | C | C | T | T | – | A | T | A |
| H5 | T | T | T | A | T | C | C | T | T | – | A | T | A |
| H6 | T | T | T | A | T | C | C | T | T | – | A | T | A |
| H7 | T | T | T | A | T | C | C | T | T | – | A | T | A |

a GenBank™ accession numbers for ITS-1 and ITS-2 sequences were AJ628043 and AJ557569, respectively

b GenBank™ accession numbers for ITS-1 and ITS-2 sequences were AJ628430 and AJ557567, respectively

c GenBank™ accession numbers for ITS-1 and ITS-2 sequences were AJ628431 and AJ557571, respectively

d GenBank™ accession number for complete ITS sequences was AM709612
