# Supplementary material for: An Outbreak of Human Fascioliasis gigantica in Southwest China
Source: PLoS One. 2013 Aug 8;8(8):e71520. doi: 10.1371/journal.pone.0071520 (PMC3738520; doi:10.1371/journal.pone.0071520)
Supplement: Table S2 — Variables included in statistical analysis. (DOC) [file pone.0071520.s003.doc]

Table S2 Variables included in statistic analysis

| **Variable code** | **Variable explanation** | **Variable value** | **P-value (univariable analysis)** | **P-value (stratified analysis)** | | | |  | **Conditional logistic regression** | | | |
| --- | --- | --- | --- | --- | --- | --- | --- | --- | --- | --- | --- | --- |
|  |  |  |  | **Q1** | **Q2** | **Q3** | **Q9** |  | **P-value** | **OR** | **Lower** | **Upper** |
| Q1 | Traveling history in the second half of 2011 | 0=no; 1=yes | 1.0000 | - | - | - | - |  |  |  |  |  |
| Q2 | Raising cattle | 0=no; 1=yes | 0.6115 | - | - | - | - |  |  |  |  |  |
| Q3 | Raising goats | 0=no; 1=yes | 0.1443 | - | - | - | - |  |  |  |  |  |
| Q4_1 | Consuming shoot with leaves of *Houttuynia cordata* in the second half of 2011 | 0=no; 1=cooked; 2=raw; 3=both | **0.0006*** | **0.0066*** | **0.0056*** | **0.0097*** | **0.0303*** |  | **0.0174*** | 2.134 | 1.142 | 3.987 |
| Q4_2 | Consuming bare root of *Houttuynia cordata* in the second half of 2011 | 0=no; 1=cooked; 2=raw; 3=both | **0.0053*** | 0.1405 | 0.1774 | 0.0904 | 0.2177 |  |  |  |  |  |
| Q5 | Consuming watercress in the second half of 2011 | 0=no; 1=cooked; 2=raw; 3=both | **0.0327*** | 0.1104 | 0.0603 | 0.119 | 0.1595 |  |  |  |  |  |
| Q6 | Consuming wildrice stem in the second half of 2011 | 0=no; 1=cooked; 2=raw; 3=both | **0.0239*** | 0.1743 | 0.123 | **0.0357*** | 0.4336 |  |  |  |  |  |
| Q7 | Consuming scallion in the second half of 2011 | 0=no; 1=cooked; 2=raw; 3=both | 0.1557 | 0.5013 | 0.391 | 0.2279 | 0.7039 |  |  |  |  |  |
| Q8 | Consuming wild vegetables in the second half of 2011 | 0=no; 1=yes | **0.0379*** | 0.2368 | 0.1152 | 0.4186 | 0.0758 |  |  |  |  |  |
| Q9 | Drinking | 0=no; 1=occasional (≦5 per year); 2=sometimes (≦2 per month);3=often (>2 per month); 4=always (>20 per month) | 0.1070 | - | - | - | - |  |  |  |  |  |

* significant difference (level=0.05)
