# Supplementary material for: An Outbreak of Human Fascioliasis gigantica in Southwest China
Source: PLoS One. 2013 Aug 8;8(8):e71520. doi: 10.1371/journal.pone.0071520 (PMC3738520; doi:10.1371/journal.pone.0071520)
Supplement: Table S3 — The profile of biochemical indicators in patients. (DOC) [file pone.0071520.s004.doc]

Table S3. The profile of biochemical indicators in patients

| **SN** | **Indicator** | **No. Patient** | **No. M*** | **No. MA**** | **Ratio** | **Range** | **Median** | **Abnormal** | **Normal** | **Variable** |
| --- | --- | --- | --- | --- | --- | --- | --- | --- | --- | --- |
| 1 | ALT | 29 | 133 | 67+ | 0.50 | 0.025-4.740 | 0.540 | 5 | 5 | 19 |
| 2 | AST | 29 | 130 | 48+ | 0.37 | 0.025-1.800 | 0.313 | 5 | 7 | 17 |
| 3 | ALP | 29 | 101 | 84+ | 0.83 | 0.007-6.143 | 1.150 | 19 | 2 | 8 |
| 4 | GGT | 29 | 98 | 93+ | 0.95 | 0.160-9.600 | 2.020 | 25 | 1 | 3 |
| 5 | CK | 29 | 88 | 60- | 0.75 | 0.038-0.792 | -0.538 | 13 | 3 | 13 |
| 6 | LDH | 29 | 109 | 68+ | 0.62 | 0.004-1.196 | 0.263 | 9 | 4 | 16 |
| 7 | HBDH | 29 | 99 | 55+ | 0.56 | 0.005-1.181 | 0.203 | 9 | 8 | 12 |
| 8 | CHE | 22 | 86 | 58- | 0.67 | 0.002-0.837 | -0.292 | 13 | 4 | 5 |
| 9 | ALB | 29 | 133 | 100- | 0.75 | 0.003-0.400 | -0.131 | 13 | 1 | 15 |
| 10 | GLO | 29 | 126 | 101+ | 0.80 | 0.003-1.147 | 0.280 | 16 | 1 | 12 |
| 11 | A/G | 29 | 126 | 124- | 0.98 | 0.026-0.713 | -0.340 | 27 | 0 | 2 |
| 12 | D-BIL | 29 | 132 | 23+ | 0.17 | 0.039-2.283 | 0.383 | 1 | 16 | 12 |
| 13 | TBA | 29 | 116 | 33+ | 0.28 | 0.010-13.880 | 0.290 | 1 | 9 | 19 |
| 14 | CRP | 29 | 90 | 87+ | 0.97 | 0.244-33.448 | 10.260 | 27 | 1 | 1 |

* The number of measures

** The number of abnormal measures

+ level increased

- level declined
