# Supplementary material for: An Outbreak of Human Fascioliasis gigantica in Southwest China
Source: PLoS One. 2013 Aug 8;8(8):e71520. doi: 10.1371/journal.pone.0071520 (PMC3738520; doi:10.1371/journal.pone.0071520)
Supplement: Table S4 — The profile of blood cellular indicators in patients. (DOC) [file pone.0071520.s005.doc]

Table S4. The profile of blood cellular indicators in patients

| **SN** | **Indicator** | **No. Patient** | **No. M*** | **No. MA**** | **Ratio** | **Range** | **Median** | **Abnormal** | **Normal** | **Variable** |
| --- | --- | --- | --- | --- | --- | --- | --- | --- | --- | --- |
| 1 | EO% | 29 | 142 | 137+ | 0.965 | 0.040-13.200 | 5.000 | 26 | 0 | 3 |
| 2 | EO# | 29 | 134 | 127+ | 0.948 | 0.136-41.680 | 6.340 | 23 | 0 | 6 |
| 3 | HGB | 29 | 160 | 143- | 0.894 | 0.009-0.592 | -0.225 | 19 | 2 | 8 |
| 4 | HCT | 29 | 160 | 143- | 0.894 | 0.018-0.530 | -0.243 | 19 | 2 | 8 |
| 5 | R-SD | 29 | 140 | 107+ | 0.764 | 0.002-0.580 | 0.141 | 15 | 4 | 10 |
| 6 | RBC | 29 | 161 | 120- | 0.745 | 0.003-0.553 | -0.206 | 14 | 4 | 11 |
| 7 | R-CV | 29 | 144 | 100+ | 0.694 | 0.006-0.506 | 0.097 | 11 | 5 | 13 |
| 8 | WBC | 29 | 161 | 105+ | 0.652 | 0.010-1.890 | 0.325 | 9 | 3 | 17 |
| 9 | PLT | 29 | 154 | 84+ | 0.545 | 0.010-0.863 | 0.177 | 5 | 4 | 20 |

* The number of measures

** The number of abnormal measures

+ level increased

- level declined
