# Supplementary material for: An Outbreak of Human Fascioliasis gigantica in Southwest China
Source: PLoS One. 2013 Aug 8;8(8):e71520. doi: 10.1371/journal.pone.0071520 (PMC3738520; doi:10.1371/journal.pone.0071520)
Supplement: Table S5 — The prevalence of fascioliasis in domestic animals. (DOC) [file pone.0071520.s006.doc]

Table S5. The prevalence of fascioliasis in domestic animals

| **Community** | **Cattle** | | |  | **Goat** | | | **Overall prevalence (%)** |
| --- | --- | --- | --- | --- | --- | --- | --- | --- |
|  | **No. total** | **No. positive** | **No. negative** |  | **No. total** | **No. positive** | **No. negative** |  |
| Xinzhuang | 46 | 15 | 31 |  | 25 | 21 | 4 | 50.7 |
| Xinping | 72 | 26 | 46 |  | - | - | - | 36.1 |
| Guantong | 25 | 9 | 16 |  | - | - | - | 36.0 |
| Zhouguan | 117 | 37 | 80 |  | 1 | 1 | 0 | 32.2 |
| Xindui | 22 | 7 | 15 |  | 3 | 1 | 2 | 32.0 |
| Daluocheng | 46 | 18 | 28 |  | 20 | 2 | 18 | 30.3 |
| Shimaping | 27 | 8 | 19 |  | - | - | - | 29.6 |
| Shuangdun | 55 | 9 | 46 |  | - | - | - | 16.4 |
| Liguan | 10 | 2 | 8 |  | 7 | 0 | 7 | 11.8 |
| Housuo | - | - | - |  | 11 | 1 | 10 | 9.1 |
| Zhoucheng | 25 | 1 | 24 |  | 10 | 1 | 9 | 5.7 |
| Maguanying | 23 | 2 | 21 |  | 20 | 0 | 20 | 4.7 |
| Liliu | - | - | - |  | 7 | 0 | 7 | 0.0 |
